# Is color continuously activated in mental simulations across a broader discourse context?

**DOI:** 10.3758/s13421-020-01078-6

**Published:** 2020-08-12

**Authors:** Lara N. Hoeben Mannaert, Katinka Dijkstra, Rolf A. Zwaan

**Affiliations:** Erasmus University Rotterdam, Burgemeester Oudlaan 50, Mandeville Building, PO Box 1738, 3000 DR Rotterdam, The Netherlands

**Keywords:** Mental simulation, Situation models, Color, Language comprehension, Grounded cognition

## Abstract

Previous studies have provided contradictory information regarding the activation of perceptual information in a changing discourse context. The current study examines the continued activation of color in mental simulations across one (Experiment 1), two (Experiment 2), and five sentences (Experiment 3), using a sentence-picture verification paradigm. In Experiment 1, the sentence either contained a reference to a color (e.g., a red bicycle) or no reference to a color (e.g., bicycle). In Experiments 2 and 3, either the first or the final sentence contained a reference to a color. Participants responded to pictures either matching the color mentioned in the sentence, or shown in grayscale. The results illustrated that color was activated in mental simulations when the final sentence contained a reference to color. When the target object (e.g., bicycle) was mentioned in all sentences (i.e., in Experiment 2), color remained activated in the mental simulation, even when only the first sentence made a reference to a color. When the focus of the story was shifted elsewhere and the target object was not present across all sentences (i.e., in Experiment 3), color was no longer activated in the mental simulation. These findings suggest that color remains active in mental simulations so long as the target object is present in every sentence. As soon as the focus of the story shifts to another event, this perceptual information is deactivated in the mental simulation. As such, there is no continued activation of color across a broader discourse context.

*He wore a tall pointed grey hat, a long grey cloak, and a silver scarf. He had a long white beard and bushy eyebrows that stuck out beyond the brim of his hat.* (Tolkien, 2005, p. 25)

Whenever a new character is introduced in a story, certain details about the appearance of the character are mentioned. In the quote above, a clear description of the character Gandalf in the *Lord of the Rings* book series is given, and immediately the reader gains a good idea of how this character would look in real life. This mental representation of Gandalf is constructed at the beginning of the novel and is maintained throughout the series. When this character changes in appearance (Gandalf the Grey becomes Gandalf the White), this mental representation is presumably updated to accommodate these changes.

Mental representations do not merely describe the superficial text structure, but are thought to contain the meaning described by a text, also known as the situation model (Van Dijk & Kintsch, 1983). According to the event indexing model, comprehenders integrate the characters and objects, goals, locations, events, and actions described in a text into a situation model (Zwaan, Langston, & Graesser, 1995; Zwaan, Magliano, & Graesser, 1995). Indeed, a plethora of studies have found evidence that multiple dimensions are tracked during language comprehension. For example, when discourse violates temporal, causal, protagonist-related, and goal-related continuity, reading times increase (Zwaan, Radvansky, Hilliard, & Curiel, 1998). Moreover, spatial information is also tracked and incorporated into the situation model (Levine & Klin, 2001), especially when a narrative forces spatial relations to be causally relevant (Sundermeier, Van der Broek, & Zwaan, 2005). Even changes in neural activity have been associated with the tracking of the temporal dimension in short texts (Ditman, Holcomb, & Kuperberg, 2008), and it is thought that memory is worse for events that preceded a time shift (Ditman et al., 2008; Speer & Zacks, 2005; Zwaan, 1996). As such, it is generally agreed upon that many dimensions are tracked during language comprehension and are incorporated into the updated situation model.

So, what happens to the activation of these dimensions as distance to the target referent is increased? Rinck and Bower (1995; see also Glenberg, Meyer, & Lindem, 1987) investigated whether spatial distance in a situation model influences anaphoric resolution and found that when spatial distance is increased, the accessibility of the referents is reduced. Thus, if an object is far away from the reader’s focus of attention, then it is harder for the reader to understand an anaphoric reference to that object. This study suggests that, although many dimensions are tracked during language comprehension, not all information is retained in a situation model throughout a narrative.

However, the question of what happens to the perceptual features of entities, such as the color of Gandalf’s cloak, throughout a narrative is still unanswered. Given that comprehenders track events throughout a narrative, do they activate all of the associated information every time a particular dimension (e.g., entity) is mentioned? Or do they only activate when a change occurs on a particular dimension? For example, if one were to read the text: “*The boy rode on the red bicycle to the station. At the station he stepped off of his bicycle*.”, would the color “red” be reactivated when the word *bicycle* is mentioned the second time, or does color become irrelevant after the introduction of the object? Relating this back to the description of Gandalf, would readers create a mental simulation of Gandalf’s appearance (including his grey or white cloak) each time the character is mentioned in the books, or are specific perceptual features irrelevant for these simulations?

Mental simulations are defined as the “reenactment of perceptual, motor, and introspective states acquired during experience with the world, body, and mind” (Barsalou, 2008, p. 618). When the concept of the situation model was first introduced, the composition of the situation model was considered to be amodal in nature. More recently, however, many researchers are of the belief that the event representations that form the situation model are actually perceptual in nature (Barsalou, 1999, 2008; Zwaan, 2016). Indeed, much research has been published that provides support for sensorimotor activation during language comprehension (see Barsalou, 2008; Dove, 2016; Kiefer & Pulvermüller, 2012, for extensive reviews on this topic). Specifically, many studies using the sentence-picture verification paradigm have found evidence that various object features are included in mental simulations, such as object shape (Zwaan, Stanfield, & Yaxley, 2002), orientation (Stanfield & Zwaan, 2001), motion (Zwaan, Madden, Yaxley, & Aveyard, 2004), visibility (Yaxley & Zwaan, 2006), and color (Hoeben Mannaert, Dijkstra, & Zwaan, 2017; Zwaan & Pecher, 2012), but these have not examined the activation of these object features over the course of more than one sentence.

So what happens to an object representation after its initial activation in a mental simulation? A study by Pecher, Van Dantzig, Zwaan, and Zeelenberg (2009) showed that comprehenders can retain the implied shape and orientation of objects for 45 minutes, suggesting that mental simulations can be reactivated when a task requires it. However, it has also been shown in several studies that when a time shift occurs, or when a character changes location, that memory is worse for events that preceded those changes (Ditman et al., 2008; Morrow, Greenspan, & Bower, 1987; Radvansky & Copeland, 2006; Speer & Zacks, 2005; Zwaan, 1996). The lack of accessibility of previous information is thought to be due to the creation of a new situation model, which is thought to clear the information from previous events from active memory (Swallow, Zacks, & Abrams, 2009). Furthermore, it has also been found that memory for perceptual information can be enhanced if the target object is present at event boundaries (Swallow et al., 2009). As such, it is still unclear what exactly occurs with perceptual information over the course of a narrative.

To our knowledge, no studies have yet looked at the continuous activation of perceptual features across a wider discourse context by using a sentence-picture verification paradigm. This paradigm is an effective method for examining the activation of perceptual information in mental simulations. If a perceptual feature is activated, participants respond significantly faster when the picture they see matches that feature, compared with when it mismatches (the so-called “match effect”). If a character or object is reintroduced later in a text, does this lead to a reactivation of the associated perceptual features? Or is fixation of a perceptual feature the only means of retaining perceptual activation in mental simulations? Arguably, in our “red bicycle” example, the color of the bicycle would not *need* to be retrieved in subsequent mentions of the bicycle for readers to maintain a clear understanding of the situation described by the text.

On the other hand, if a comprehensive situation model is built at each section of a narrative, you would expect all relevant information to become reactivated at each mention of the object or character. Support for this assertion comes from the fact that comprehenders retain perceptual information for long periods of time (Pecher et al., 2009). As such, there appear to be contradictory theories and studies regarding what happens to perceptual information during discourse processing, which is the focus of the current study.

## The current study

Supporters of the grounded cognition view argue that the simulation system is required for language comprehension. The exact mechanism, however, is still unclear. According to the language and situated simulation (LASS) theory, the linguistic system is activated first during language comprehension, and only if this system is insufficient for complete comprehension does the simulation system activate (Barsalou, Santos, Simmons, & Wilson, 2008). As mentioned previously, when object features are implied in a sentence, the simulation system activates. The question is whether this also occurs if the object feature is no longer relevant to the story. To our knowledge, only two studies have examined the activation of the sensorimotor system in a wider discourse context.

A study by Hoeben Mannaert, Dijkstra, and Zwaan (2019) found that over the course of two or four sentences, when a change in shape is implied, mental simulations are updated by replacing the initial simulation with the changed shape. Furthermore, a study by Zheng, Huang, Zhong, Li, and Mo (2017) found that when participants hear narratives continuously referring to the color green, they were more likely to see a red square on a white background. This linguistic adaptation color aftereffect is the same as the perceptual adaptation color aftereffect (i.e., staring at a green square for several minutes creates a red afterimage when viewing a blank page directly afterwards), suggesting that mental simulations of color use the same neural substrates as color perception. However, both of these studies still actively refer to the object feature of interest throughout the narratives. As such, it can only be concluded that the simulation system is activated when the object feature is referred to in the discourse. What happens to this activation once this reference is eliminated remains unclear.

To further our understanding of the underlying mechanisms of mental simulations in language comprehension, it is important that we know what the role of perceptual information is in mental simulations, and whether there is continued activation of this perceptual information. We conducted three experiments to examine this. Each experiment used a sentence-picture verification paradigm, where participants read sentences that described an object in combination with (or without) a color, followed by a picture that had to be verified. The picture either matched the color mentioned in the sentence or was shown in grayscale. In Experiment 1, participants viewed only one sentence before they saw the picture, the sentence therefore either containing one reference to a colored object or containing a reference only to the object, without color (see Table 1). Based on the studies that have shown that participants respond significantly faster when pictures match the color implied in a text (Hoeben Mannaert et al., 2017; Zwaan & Pecher, 2012), we expect that participants will respond significantly faster to pictures shown in color compared with grayscale for sentences that make explicit reference to color. For sentences that contain no reference to color, we expected to find no difference in response times, as the items we used were low in color diagnosticity (Tanaka & Presnell, 1999).

**Table 1:**
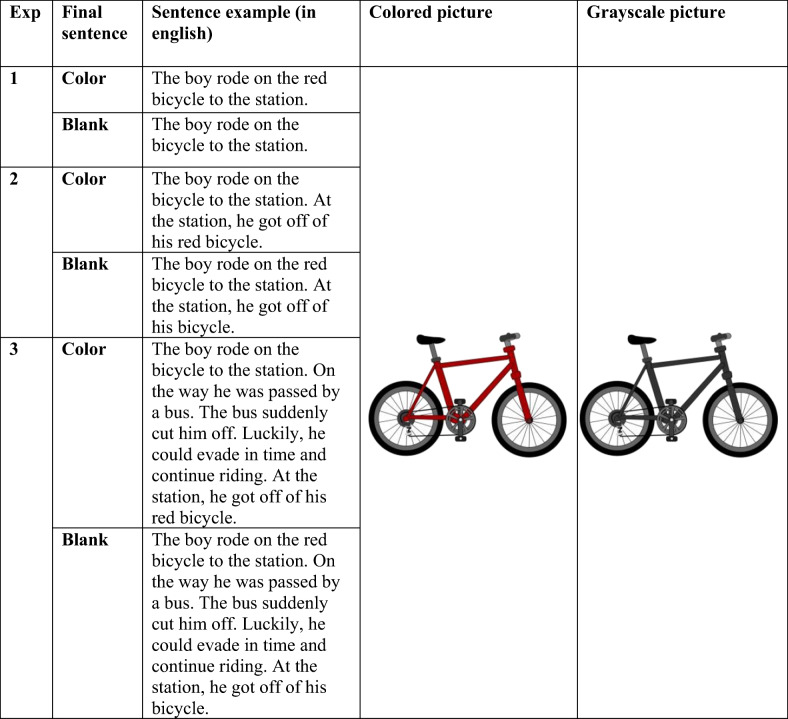
Example of a sentence item and a picture item for each experiment

*Note.* The examples provided here are in English, but the study used Dutch sentences. The translation therefore may not be exact

Experiment 2 was an extension of Experiment 1 as here participants read two sentences, where either the first or the last sentence contained a reference to color. Given that color would no longer be referred to in the second sentence, we expected to find no significant difference between the colored pictures and the grayscale pictures when only the first sentence contained a reference to color. Similar to Experiment 1, we did expect to find a facilitation effect where participants responded significantly faster to the colored picture compared with the grayscale picture when the final sentence contained a reference to color.

For Experiment 3 we constructed stories in which the focus was shifted away from the target object for several sentences. Participants read five sentences before responding to the picture, where either the first or the final sentence contained a reference to color, while the middle three sentences were fillers that served to maintain coherence of the narrative, but were intended to shift attention away from the target object (see Table 1). We expected that, even when color is not mentioned in the final sentence, that participants will still show a facilitation effect, responding faster to colored pictures compared with grayscale pictures, in both sentence conditions (i.e., when the first or the final sentence contains a reference to color).

## Ethics statement

The participation in all experiments was voluntary. The participants subscribed to the experiments online via the university platform and were told that by signing up for a study they declare to voluntarily participate in this study. They were briefed with the content of each study and provided written consent. Participants were told they were free to terminate the experiment at any point in time without experiencing negative consequences. This study was approved by the Ethics Committee of Psychology at the Erasmus University Rotterdam, The Netherlands.

## Preregistration

The predictions, exclusion criteria, design, methods, analyses, and materials of all the experiments reported in this article were preregistered in advance of data collection and analysis on the Open Science Framework (OSF) to ensure confirmatory procedures were conducted according to a priori criteria. The preregistration for Experiments 1 and 2 can be viewed on https://osf.io/2nup7, the preregistration for Experiment 3 can be viewed on https://osf.io/bfm6p. Analyses that were not preregistered are referred to in this article under the heading “Exploratory Analyses.”

## Experiment 1

### Method

#### Norming study

As we were interested in testing the activation of color, it was important that the items we used were low in color diagnosticity (Tanaka & Presnell, 1999). For example, the word *pumpkin* is highly associated with the color orange; therefore, even if the word *orange* is not included in the sentence, participants would still respond faster to a picture of an orange pumpkin compared with a grayscale pumpkin, even without a color reference (Therriault, Yaxley, & Zwaan, 2009). To ensure that the findings from our study could not be confounded by effects of color diagnosticity, we performed a norming study to control for this. As such, we created a list of items that were partially taken from the low color diagnosticity items in the Tanaka and Presnell (1999) and Nagai and Yokosawa (2003) studies. As we needed more items than the ones used by those studies, we created the remainder of the stimuli ourselves. Thirty-nine Dutch first-year bachelor’s students at the Erasmus University Rotterdam (35 females, age range: 17–26 years) took part in the norming study. Participants performed a word-picture verification task, where they first saw the word in the center of the screen, followed by a picture that was either shown in color or in grayscale. Forty-eight experimental items were shown in grayscale and in color (resulting in 96 experimental items shown in total), and 48 filler items were also shown in grayscale and in color (resulting in 96 filler items shown in total). Participants were instructed to respond “yes” (the “L” key) when the picture matched the preceding word, and were instructed to respond “no” (the “A” key) when the picture did not match. A paired-samples *t* test found no significant color advantage in the response times for either experimental items, *t*_*1*_(38) = 0.06, *p* = .956; *t*_*2*_(47) = 0.03, *p* = .980, or for filler items, *t*_*1*_(38) = 1.80, *p* = .091; *t*_*2*_ (47)= 0.17, *p* = .864. Accuracy scores also showed no significant color advantage for either experimental items, *t*_*1*_(38) = 0.89, *p* = .378; *t*_*2*_(47) = 0.92, *p* = .361, or filler items, *t*_*1*_(38) = 0.42, *p* = .680; *t*_*2*_(47) = 0.54, *p* = .595. As such, the items used in the current study show no evidence of having high color diagnosticity.

#### Participants

A power analysis was done using the results of Experiment 1 from Hoeben Mannaert et al. (2017), which used a similar paradigm to test whether color is represented in mental simulations. With an effect size of *f* = 0.13, it was calculated that a minimum of 82 participants would be required to find an effect if there is one (α = .05, power = .80). To ensure our study had sufficient power after potential exclusions, 100 Dutch psychology students (77 females, *M*_*age*_ *=* 20.79 years, *SD*_age_ = 3.07 years) from the Erasmus University Rotterdam were recruited to take part in Experiment 1. Participants were excluded if they had a total accuracy percentage of 80% or less, which led to the exclusion of five participants, resulting in a sample of 95.

#### Materials

One hundred and ninety-two sentences were created that either included a reference to color (96 sentences) or omitted any reference to color (96 sentences). Of these sentences, half (96 sentences) were used as filler sentences, meaning that the picture shown afterwards did not match the object described in the sentence; the other half were experimental sentences. Given that each object was described by both a sentence containing a reference to color and a sentence containing no reference to color, each participant received only one version of these sentences, meaning that each participant read 48 experimental sentence items and 48 filler sentence items. Similarly, they saw 48 experimental pictures and 48 filler pictures, which were found using the Google search engine and edited using the Paint.NET software (Version 4.1.5), were either depicted in the color matching the sentence or in grayscale, and did not exceed a 300 × 300 pixel resolution (approximately 7.9 × 7.9 cm on screen). In total, participants received 96 sentence items and 96 pictures. Additionally, participants received 24 comprehension questions to check whether they properly read the sentences. An example of the sentence items and pictures used in the current study can be seen in Table 1.

The experiment was programmed using E-Prime 2.0 Professional, and participants completed the experiments in isolated cubicles with computers equipped with 24.1-in. TFT-IPS screens with a resolution of 1,920 × 1,200 and a ratio of 16:10.

#### Design

The experiment is a 2 (sentence: color vs. blank) × 2 (picture: color vs. grayscale) within-subjects design. Four lists were constructed to ensure sufficient counterbalancing, so that a sentence could either include a color referral or not, and that a picture could either be shown in color or in grayscale. An additional experiment from another study was performed by the participants in the same session, which was counterbalanced to be completed either before or after the current study; experiment order did not influence the results from the current study.

#### Procedure

Participants were instructed that they would perform a self-paced reading task using the space bar and that they would see a picture after each sentence that either did represent the object described in the sentence or did not. They were instructed to respond to the shape of the object and not to the color. If the picture matched the object in the sentence, they had to respond “yes” using the “L” key, and if it did not match then they had to respond “no” using the “A” key. Half of all filler items were followed by a comprehension question, which were closed questions requiring a “yes” or “no” response. The purpose of the comprehension question was to ensure that participants properly read the sentences, rather than simply the object of the sentence. Before starting the experiment, they received six practice items.

A trial looked as follows: Participants saw the “>” symbol left aligned in the center of the screen for 1,000 ms. Subsequently, the sentence was shown left aligned in the center of the screen and remained on-screen until participants pressed the space bar. Subsequently, a fixation cross appeared in the center of the screen (center aligned) for 500 ms, after which the image appeared in the center of the screen (center aligned) and remained on-screen until participants provided a response.

### Results

#### Data analysis

A repeated-measures analysis of variance (rmANOVA) was run on the data, using “sentence version” and “picture version” as repeated-measures variables. “List” was used as a between-subjects variable to improve the quality and power of our analyses (Pollatsek & Well, 1995). All response-time analyses were performed on correct responses only. Per participant, the median response time was taken per condition, as is common in sentence-picture verification studies (Hoeben Mannaert et al., 2017, 2019; Zwaan & Pecher, 2012; Zwaan et al., 2002) to prevent extreme values from influencing the data. Subject analyses are denoted with the subscript 1, and item analyses are denoted with the subscript 2. As preregistered, we conducted rmANOVAs on accuracy scores and on response times. On suggestion by the editor and reviewers, additional exploratory analyses were performed. For each experiment, a linear mixed-effects model was performed on the reaction-time data and a logistic mixed-effects model on the accuracy data. These exploratory analyses can be found in Appendix 1. Additionally, Bayes factors (BF) were calculated for all analyses on RTs, and were analyzed using JASP (Version 0.12.2).

#### Accuracy

The rmANOVA performed on the accuracy scores illustrated a significant effect of “sentence” in both the subject and item analyses, *F*_*1*_(1, 91) = 4.41, *p* = .039; *F*_*2*_(1, 47) = 4.78, *p* = .034, where participants scored significantly more accurately on sentences that contained references to color (*M* = .99, *SE* = .003) compared with sentences that did not contain a reference to color (*M* = .98, *SE* = .003). There was a significant effect of “picture” only in the item analyses, *F*_*1*_(1, 91) = 2.52, *p* = .116; *F*_*2*_(1, 47) = 5.99, *p* = .018, where participants responded significantly faster when the picture was shown in color (*M* = .98, *SE* = .003) compared with when it was shown in grayscale (*M* = .98, *SE* = .004). Similarly, there was only a significant interaction between “sentence” and “picture” in the item analysis, *F*_*1*_(1, 91) = 3.40, *p* = .069; *F*_*2*_(1, 47) = 5.77, *p* = .020. There was a significant interaction between “list” and “picture.” *F*_*1*_(3, 91) = 2.87, *p* = .041. The logistic mixed effects analysis (see Appendix 1) revealed only a significant main effect of “sentence,” χ^2^(1) = 4.60, *p* = .032, and “picture,” χ^2^ (1) = 4.37, *p* = .037, but no significant interaction effect between “sentence” and “picture,” χ^2^(1) = 2.33, *p* = .127. Given that the logistic mixed effects model includes both subject and item analyses, it is more likely that for the accuracy scores there is only a main effect of “picture” and “sentence.” However, as these percentage differences are not larger than 1% between conditions, it is not meaningful to interpret them.

#### Exploratory analyses (accuracy)

A paired-samples *t* test showed that participants responded significantly more accurately to the colored picture (*M* = .98, *SD* = .04) than to the grayscale picture (*M* = .97, *SD* = 06) when the sentence made reference to a color, *t*_*1*_(94) = 2.16, *p* = .033, *d* = 0.22; *t*_*2*_(47) = 2.78, *p* = .008. There was no significant difference in accuracy scores between the colored picture (*M* = .99, *SD* = .04) and the grayscale picture (*M* = .98, *SD* = .04) when the sentence contained no reference to color, *t*_*1*_(94) = 0.15, *p* = .880, *d* = 0.02; *t*_*2*_(47) = 0.08, *p* = .936.

#### Exploratory analyses (comprehension accuracy)

Analysis of the comprehension accuracy scores revealed an overall high comprehension accuracy (*M* = .93, *SD* = .12), suggesting that readers properly read the sentences in the experiment.

#### Response times

The rmANOVA performed on response times illustrated a significant effect of “sentence,” but only in the item analysis, *F*_*1*_(1, 91) = 1.55, *p* = .216; *F*_*2*_(1, 47) = 5.00, *p* = .030, where participants responded significantly faster to the sentence not referring to a color (*M* = 847.43 ms, *SE* = 31.58 ms) compared with the sentence referring to a color (*M* = 867.74 ms, *SE* = 31.18 ms). The model-averaged BF (across matched models) for “sentence” is 0.26, meaning that the data is 0.26 times more likely with “sentence” as a predictor than without. Furthermore, both subject and item analyses showed a significant effect of “picture,” *F*_*1*_(1, 91) = 9.80, *p* = .002; *F*_*2*_(1, 47) = 28.63, *p* < .001, where participants responded significantly faster to the picture shown in color (*M* = 838.69 ms, *SE* = 31.06 ms) compared with the picture shown in grayscale (*M* = 876.48 ms, *SE* = 30.73 ms). The model-averaged BF for “picture” is 2.76. Furthermore, a significant interaction between “sentence” and “picture” was found, *F*_*1*_(1, 91) = 16.10, *p* < .001; *F*_*2*_(1, 47) = 11.72, *p* = .001. The model-average BF for this interaction is 191.32. The linear mixed-effects analyses (see Appendix 1) revealed that “sentence” did not significantly improve the model fit, χ^2^(1) = 2.00, *p* = .157, and thus supports the lack of a significant effect in subject analyses of the rmANOVAS for this variable. Both “picture,” χ^2^(1) = 12.75, *p* < .001, and the interaction between “picture” and “sentence,” χ^2^(2) = 25.01, *p* < .001, however, did significantly improve model fit and fall in line with the findings from the rmANOVAs.

#### Exploratory analyses (response times)

**Fig. 1:**
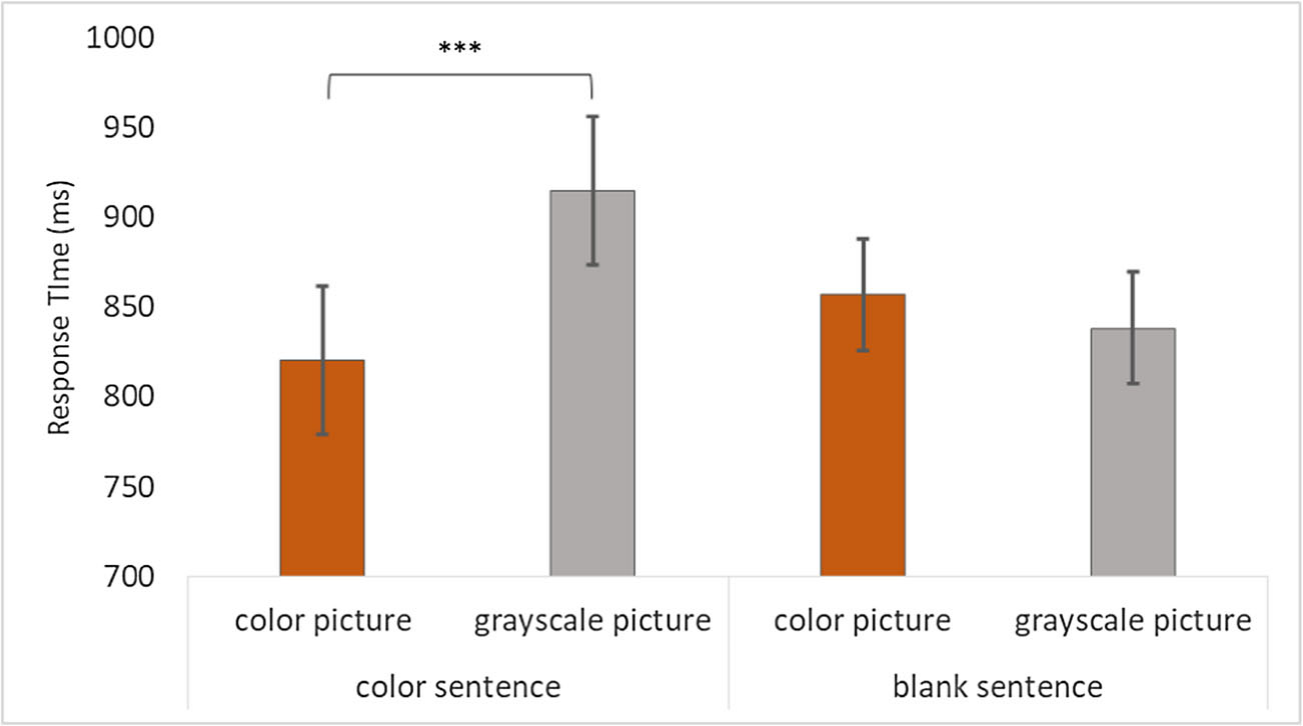
Bar graph displaying the average response times per condition for Experiment 1. Error bars show 95% CI. ***p < .001

A paired-samples *t* test was conducted to examine the interaction between “sentence” and “picture,” and found that participants responded significantly faster to the colored picture (*M* = 821 ms, *SD* = 324 ms) than to the grayscale picture (*M* = 915 ms, *SD* = 323 ms) when the sentence contained a reference to a color, *t*_*1*_(94) = −4.48, *p* < .001, *d* = −0.46; *t*_*2*_(47) = −5.14, *p* < .001 (see Fig. 1). There was no significant difference between the colored picture (*M* = 857 ms, *SD* = 321 ms) and the grayscale picture (*M* = 838 ms, *SD* = 307 ms) when the sentence did not contain a reference to color, *t*_*1*_(94) = −1.17, *p* = .245, *d* = −0.12; *t*_*2*_(47) = −0.56, *p* = .578.

### Discussion

As predicted, there was a significant color advantage when the sentence contained a reference to color, while no such advantage was present when the sentence did not contain a reference to a color. Although both the accuracy and the response-time analyses support this conclusion, it should be noted that accuracy scores overall were very high (between 97% and 99% across conditions). Given that the significant difference in the color condition is only a difference of 1%, this is not very meaningful.

Experiment 1 has established that color is activated in mental simulations when it is mentioned for the first time, and thus supports the findings of previous studies on color simulation (e.g., Hoeben Mannaert et al., 2017; Zwaan & Pecher, 2012). Experiment 2 serves to expand on this finding by examining whether this activation remains if participants read two sentences, where either the first or the final sentence contain a reference to color.

## Experiment 2

The aim of Experiment 2 was to examine whether the activation of color in mental simulations would change across two sentences. In the current experiment, participants read sentences where either the first sentence contained a reference to a color, or the final sentence contained a reference to a color (see Table 1). We predicted that color would have deactivated if the second sentence made no reference to color, and in that condition expected to find no significant difference in response times between the colored picture and the grayscale picture. If color would not have deactivated by the second sentence, then we would expect to find a significant color advantage, similar to what was found in Experiment 1. For the condition where the final sentence contained a reference to color, we did expect to find a significant difference between the colored picture and the grayscale picture.

### Method

#### Participants

One hundred Dutch psychology students (77 females, *M*_age_ *=* 20.47 years, *SD*_age_ = 3.34 years) from the Erasmus University Rotterdam were recruited to take part in the current study. Participants were excluded if they had a total accuracy percentage of 80% or less; as a result, of this exclusion criteria, three participants were excluded from the analysis. The final sample consisted of 97 participants.

#### Materials

The sentences from Experiment 1 were expanded to contain two sentences per item (see Table 1 for an example). The sentences either contained a reference to color in the first sentence or in the second sentence. The rest of the materials were identical to Experiment 1.

#### Design and procedure

The design and procedure of Experiment 2 was identical to Experiment 1, except that participants were informed that they would see the picture after every two sentences.

### Results

#### Data analysis

The same analysis plan used for Experiment 1 was also used for Experiment 2.

#### Accuracy

The rmANOVA for accuracy scores revealed a significant effect of “picture,” *F*_*1*_(1, 93) = 5.97, *p* = .016; *F*_*2*_(1, 47) = 4.37, *p* = .042, where participants responded significantly faster when the pictures were shown in color (*M* = .99, *SE* = .002) compared with when they were shown in grayscale (*M* = .98, *SE* = .003). However, there was no significant effect of “sentence,” *F*_*1*_(1, 93) = 0.0007, *p* = .980; *F*_*2*_(1, 47) = .001, *p* = .972, nor a significant interaction between “sentence” and “picture,” *F*_*1*_(1, 93) = 1.51, *p* = .223; *F*_*2*_(1, 47) = 0.53, *p* = .470. “List” interacted significantly with “picture,” *F*_*1*_(3, 93) = 14.61, *p* < .001. The logistic mixed effects analysis (see Appendix 1) also only found a main effect of “picture,” χ^2^(1) = 6.36, *p* = .012, but no significant effect of “sentence,” χ^2^(1) < 0.001, *p* = .981, nor a significant interaction effect between “sentence” and “picture,” χ^2^(2) = 1.36, *p* = .506.

#### Exploratory analyses (accuracy)

A paired-samples *t* test illustrated that participants responded significantly more accurately to the colored pictures (*M* = .99, *SD* = .03) compared with the grayscale pictures (*M* = .97, *SD* = .04) when the final sentence made a reference to color, but this was not significant in the item analysis, *t*_*1*_(96) = 2.48, *p* = .015, *d* = 0.25; *t*_*2*_(47) = 1.96, *p* = .056. There was no significant difference in accuracy scores between the colored picture (*M* = .98, *SD* = .04) and the grayscale picture (*M* = .98, *SD* = .04) when the first sentence made a reference to color, *t*_*1*_(96) = 0.87, *p* = .389, *d* = 0.09; *t*_*2*_(47) = 0.68, *p* = .497.

#### Exploratory analyses (comprehension accuracy)

Analysis of the comprehension accuracy scores revealed an overall high comprehension accuracy (*M* = .89, *SD* = .18), suggesting that readers properly read the sentences in the experiment.

#### Response times

The rmANOVA for response times yielded a significant main effect of “picture,” *F*_*1*_(1, 93) = 20.07, *p* < .001; *F*_*2*_(1, 47) = 39.40, *p* < .001, where participants responded significantly faster when the picture was shown in color *(M* = 829.00 ms, *SE* = 29.57 ms) compared with when it was shown in grayscale *(M* = 883.80 ms, *SE* = 28.25 ms). The model-averaged BF (across matched models) for “picture” was 8,287.40, meaning that the current data were 8,287.40 times more likely when “picture” was included as a predictor compared with when it was excluded. There was no significant effect of “sentence,” *F*_*1*_(1, 93) = 2.90, *p* = .092; *F*_*2*_(1, 47) = 0.182, *p* = .672. The model-averaged BF for “sentence” was 0.52. There was also no significant interaction between “sentence” and “picture,” *F*_*1*_(1, 93) = 0.31, *p* = .580; *F*_*2*_(1, 47) = 1.07, *p* = .307. The model-averaged BF for this interaction was 0.15. There was a significant interaction between “sentence” and “list,” *F*_*1*_(3, 93) = 4.33, *p* = .007. The linear mixed-effects model (see Appendix 1) also only found a main effect of “picture,” χ^2^(1) = 17.54, *p* < .001, but no effect of “sentence,” χ^2^(1) = 0.70, *p* = .402, nor a significant interaction between “sentence” and “picture,” χ^2^(2) = 1.27, *p* < .529.

#### Exploratory analyses (response times)

**Fig. 2:**
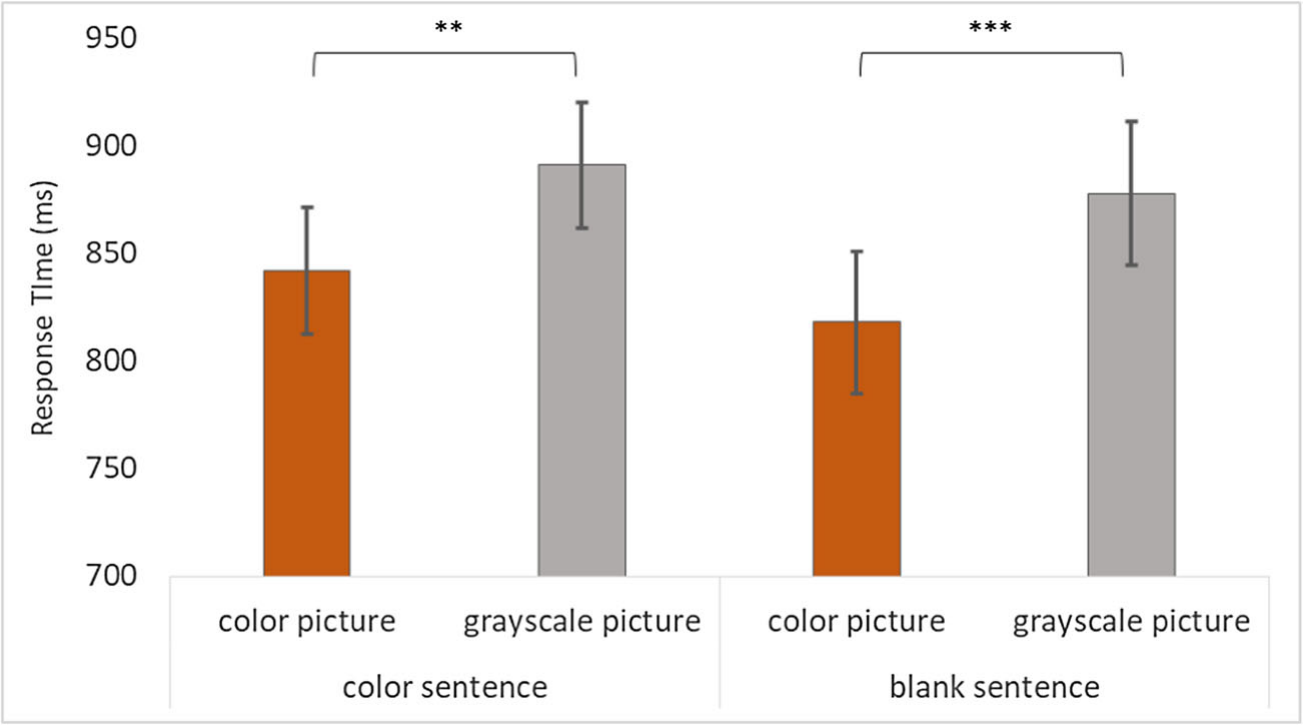
Bar graph displaying the average response times per condition for Experiment 2. “Color sentence” refers to when the final sentence referred explicitly to a color. “Blank sentence” refers to when the final sentence did not make a reference to a color, but the first sentence did. Errors bars show 95% CI. **p < .01. ***p < .001

A paired-samples *t* test on response times found that participants responded significantly faster to the colored picture (*M* = 842 ms, *SD* = 309 ms) compared with the grayscale picture (*M* = 891 ms, *SD* = 276 ms) when the final sentence made a reference to color, *t*_*1*_(96) = −3.27, *p* = .002, *d* = −0.33; *t*_*2*_(47) = −4.23, *p* < .001. Participants also responded significantly faster to the colored picture (*M* = 818 ms, *SD* = 285 ms) than to the grayscale picture (*M* = 878 ms, *SD* = 295 ms) when the first sentence made a reference to color, *t*_*1*_(96) = −3.54, *p* < .001, *d* = −0.36; *t*_*2*_(47) = −2.82, *p* = .007 (Fig. 2).

### Discussion

We had predicted that color would have deactivated when the second sentence makes no reference to a color. Interestingly, the results showed that color had remained activated, regardless of whether color was mentioned in the first or the final sentence, as participants responded significantly faster to the colored picture compared with the grayscale picture. This suggests that, when a color is first mentioned, it becomes active in mental simulations, and remains active even in the following sentence.

## Experiment 3

The aim of Experiment 3 was to examine how the activation of color would change in a wider discourse context. Participants in the current experiment read five sentences, where either the first or the final sentence contained a reference to color (see Table 1). The middle three sentences were filler sentences that maintained coherence within the story, but referred to objects or events other than the target object in the first and final sentences. Examining the activation of color in mental simulations using several sentences allowed us to examine how this activation behaves in a more naturalistic context. Based on the findings from Experiment 2, we expected to continue to find a significant color advantage, regardless of whether color was mentioned in the first or final sentence in the texts.

### Method

#### Participants

One hundred Dutch psychology bachelor’s students were recruited from the Erasmus University Rotterdam (85 females, *M*_age_ = 19.93 years, *SD*_age_ = 2.01 years). Four participants were excluded due to having an average accuracy below 80%, leaving us with a sample of 96 participants.

#### Design and procedure

The design and procedure was the same as Experiments 1 and 2, except that participants read five sentences before seeing a picture. No other study was conducted before or after this experiment.

### Results

#### Analysis plan

The same analysis plan was used as in Experiments 1 and 2.

#### Accuracy

The rmANOVA revealed a significant interaction between “sentence” and “picture,” but only in the item analyses, *F*_*1*_(1, 92) = 3.73, *p* = .057; *F*_*2*_(1, 47) = 4.77, *p* = .034. There was no significant main effect of “sentence,” *F*_*1*_(1, 92) = 0.03, *p* = .867; *F*_*2*_(1, 47) = 0.02, *p* = .889, or “picture,” *F*_*1*_(1, 92) = 2.68, *p* = .105; *F*_*2*_(1, 47) = 3.22, *p* = .079. There was a significant interaction between “list” and “picture,” *F*_*1*_(3, 92) = 3.58, *p* = .017. The results from the logistic mixed-effects model, on the other hand, found only a significant main effect of “picture,” χ^2^(1) = 4.26, *p* = .039, but no effect of “sentence,” χ^2^(1) = 0.02, *p* = .875, nor a significant interaction between “sentence” and “picture,” χ^2^(2) = 4.00, *p* = .135. Given that the logistic mixed-effects model combines both subject and item analyses, it is more likely that there was only a main effect of “picture” for the accuracy scores. However, as the average percentage difference between conditions was only 1%, the interpretation of this difference is not very meaningful.

#### Exploratory analyses (accuracy)

A paired-samples *t* test found that participants responded significantly more accurately when the picture shown was colored (*M* = .99, *SD* = .03) compared with when it was shown in grayscale (*M* = .97, *SD* = .07), when the final sentence made a reference to a color, *t*_*1*_(95) = 2.07, *p* = .041, *d* = 0.21; *t*_*2*_(47) = 3.21, *p* = .002. There was no significant difference between the colored picture (*M* = .98, *SD* = .04) and the grayscale picture (*M* = .98, *SD* = .05) when the first sentence referred to a color, *t*_*1*_(95) = 0.15, *p* = .880, *d* = 0.02; *t*_*2*_(47) = 0.09, *p* = .929.

#### Exploratory analyses (comprehension accuracy)

Analysis of the comprehension accuracy scores revealed an overall high comprehension accuracy (*M* = .88, *SD* = .10), suggesting that readers properly read the sentences in the experiment.

#### Response time

The rmANOVA revealed a significant interaction between “sentence” and “picture” in the subject analyses, *F*_*1*_(1, 92) = 17.21, *p* < .001, but not in the item analyses, *F*_*2*_(1, 47) = 2.95, *p* = .092. The model-averaged BF (across matched models) for this interaction was 230.58, meaning that this data were 230.58 times more likely when Sentence × Picture was used as a predictor compared with when it was not. There was no significant main effect of “sentence,” *F*_*1*_(1, 92) = 1.77, *p* = .187; *F*_*2*_(1, 47) = 0.0001, *p* = .992. The model-averaged BF for “sentence” was 0.15. There was also no significant main effect of “picture,” *F*_*1*_(1, 92) = 0.96, *p* = .329, *F*_*2*_(1, 47) = 1.01, *p* = .321. The model-averaged BF for “picture” was 0.17. There was a significant interaction between “list” and “sentence,” *F*_*1*_(3, 92) = 10.52, *p* < .001. A linear mixed-effects analysis (see Appendix 1) shows support for this finding, as only a significant interaction between “sentence” and “picture” was found, χ^2^(3) = 11.12, *p* = .011, but no significant main effects of “sentence,” χ^2^(1) = 0.01, *p* = .911, or “picture,” χ^2^(1) = 0.52, *p* = .472.

#### Exploratory analyses (response time)

**Fig. 3:**
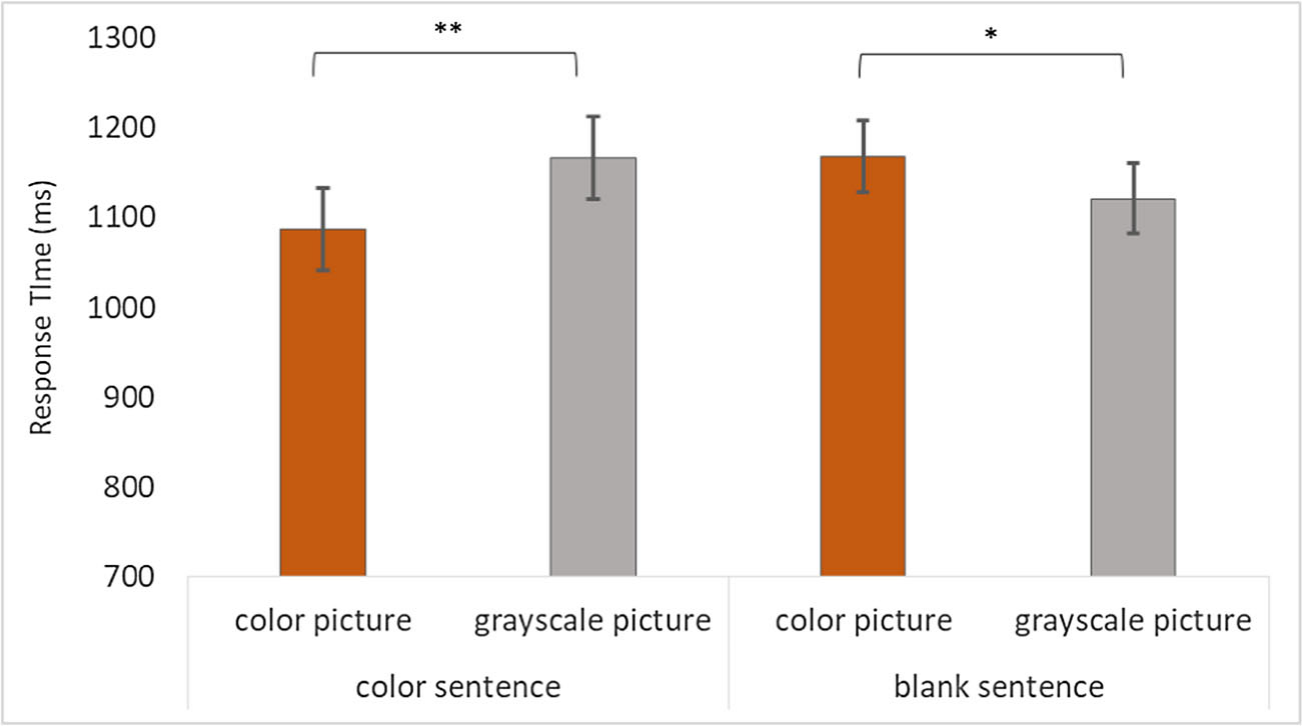
Bar graph displaying the average response times per condition for Experiment 3. “Color sentence” refers to when the final sentence referred explicitly to a color. “Blank sentence” refers to when the final sentence did not make a reference to a color, but the first sentence did. Errors bars show 95% CI. *p < .05. **p < .01

A paired-samples *t* test was performed to examine the interaction between “sentence” and “picture.” The results from the *t* test showed that participants responded significantly faster to the colored picture (*M* = 1,087 ms; *SD* = 368 ms) compared with the grayscale picture (*M* = 1,167 ms; *SD* = 407 ms) when the final sentence made a reference to color, *t*_*1*_(95) = −3.38, *p* = .001, *d* = −0.35; *t*_*2*_(47) = −1.92, *p* = .061. When the first sentence contained a reference to color, the opposite pattern emerged. Participants responded significantly faster when the picture was shown in grayscale (*M* = 1,122 ms; *SD* = 382 ms) compared with when it was shown in color (*M* = 1,169 ms; *SD* = 445 ms), *t*_*1*_(95) = 2.33, *p* = .022, *d* = 0.24, but this was not significant in the item analyses *t*_*2*_(47) = 0.87, *p* = .388 (Fig. 3).

### Discussion

Consistent with Experiments 1 and 2, accuracy was very high across all conditions, and even though the participants were significantly more accurate when responding to the colored pictures compared with the grayscale pictures when the final sentence made a reference to a color, this difference was only 2%. Given that this difference is so small, it is questionable whether such a difference is meaningful.

Similar to the previous experiments (see Fig. 1 and Fig. 2), when the final sentence contained a reference to color, color became activated in the mental simulations, as seen by the faster responses to the colored pictures compared with the grayscale pictures.

Contrary to our expectations, the analyses showed that participants do not respond faster to the colored pictures compared with the grayscale pictures when the first sentence referred to a color. In fact, the effect was reversed: Participants responded significantly faster to the grayscale picture compared with the colored picture, suggesting that color now caused interference, rather than the facilitation observed in the previous experiments.

These findings suggest that when participants read a short text where the first sentence refers to a color, the color is deactivated when attention is distracted from the target object. The interference caused by seeing the colored picture suggests that the shown image does not match the mental simulation activated during the final sentence.

## General discussion

The aim of the current study was to examine the continued activation of color in mental simulations across a wider discourse context, as much contradictory information existed regarding the perceptual activation when changes occur to a situation model. Three experiments were conducted to test this using a sentence-picture verification paradigm. Experiment 1 examined the activation of color using a single sentence, Experiment 2 used two sentences, and Experiment 3 used five sentences.

Based on the findings of previous color simulation studies (Hoeben Mannaert et al., 2017; Zwaan & Pecher, 2012), we had expected to find a color advantage in Experiment 1 when sentences referred to a color (e.g., *“The boy rode on the red bicycle to the station.”*). Indeed, participants responded significantly faster to the colored picture compared with the grayscale picture when sentences contained a reference to color. When no color reference was given (*“The boy rode on the bicycle to the station.”*), there was no significant difference in response times between the colored and grayscale pictures. This experiment provided further support for color being activated in mental simulations when a reference is made to color.

Based on the findings by Swallow et al. (2009), we had expected to find that this color advantage would disappear when two sentences are provided, when the second sentence does not refer to a color (e.g., *“The boy rode on the red bicycle to the station. At the station he stepped off of his bicycle*.*”*). Contrary to our expectations, Experiment 2 continued to show this color advantage. Participants responded significantly faster to the colored picture compared with the grayscale picture, regardless of whether the first or the final sentence contained a reference to color. The results from this experiment suggests that color continues to be active in mental simulations when only two sentences are provided. This result falls in line with the conclusions made by Swallow et al. (2009), whose findings suggested that perceptual information can remain activated in event models when objects are salient and present at event boundaries. Although event boundaries were not manipulated in the current study, it is possible that simply by mentioning the target object in both sentences, the color activation is carried over to the final sentence.

Linking this back to the updating mechanism proposed by the event indexing model, it is possible that readers use incremental updating to update their situation model in this experiment, given that the event described by the two sentences can be considered to be ongoing, and thus carry over the perceptual information across the sentences.

In Experiment 3, we expected to continue to find this color advantage as seen in Experiment 2, when participants would read five sentences. Specifically, we expected that participants would respond faster to colored pictures compared with grayscale pictures when either the first or final sentence made a reference to a color. Importantly, in this experiment the middle three sentences did not explicitly refer to the target object (e.g., *“The boy rode on the red bicycle to the station. On the way he was passed by a bus. The bus suddenly cut him off. Luckily, he could evade in time and continue riding. At the station he stepped off of his bicycle.”*). In this way, we could examine whether the perceptual information would become deactivated in the mental simulation in a more naturalistic discourse context.

Interestingly, the findings from Experiment 3 were the exact opposite to our expectations, as participants responded faster to the grayscale picture compared with the colored picture, when the first sentence made a reference to a color. This might suggest that the colored picture caused interference by it not matching up with the object activated in the mental simulation. Moreover, it would mean that color becomes deactivated over time as the focus of the narrative shifts to other objects. As other objects become incorporated into the situation model, it is possible that the perceptual features associated with the target object are no longer carried across the sentences. However, the perceptual feature “color” used in the current study was, firstly, explicitly mentioned, and, secondly, not strongly associated with the target objects. We had explicitly decided to not include any objects with a high color association (i.e., color diagnosticity). For example, items such as a pumpkin activate the color orange in a mental simulation automatically (Tanaka & Presnell, 1999). In our study, however, only items low in color diagnosticity were included (such as a bicycle), as we wanted to see whether the inclusion of a perceptual feature in a story would be carried across the narrative and reactivated whenever the target object was mentioned. As such, it is possible (and likely) that sentences including items with strong color associations continue to activate color in mental simulations throughout a narrative. This would be interesting to examine in future studies.

Furthermore, the response times of Experiment 3 are longer than those in Experiments 1 and 2. Given that color was irrelevant to the response (they had to respond to the shape of the objects) in all experiments, it is unlikely that this is the cause for the increased response times in Experiment 3. Furthermore, it is also unlikely to be due to a longer search through memory to enhance accuracy, as the target object was always mentioned in the final sentence. It is possible, however, that the increased response times are due to the building of a more elaborate situation model. In Experiment 3, several objects are being referred to and need to be incorporated into the situation model. It is likely that this integration process took longer in comparison to the first two experiments, where only one object was referred to.

Two important conclusions can be drawn from this study. Firstly, perceptual information becomes active in mental simulations when they are referred to (Experiment 1), even over the course of two sentences (Experiment 2). When attention is shifted away from the target object (Experiment 3), the perceptual information (i.e., color) no longer remains active in the mental simulation. Secondly, these findings suggest that a complete situation model, containing all related information, is not created during discourse processing. Only information that is required for language comprehension needs to be activated in the situation model. This study is the first to examine the role of mental simulations over the course of five sentences and how the activation of associated perceptual information is carried through the text when they are no longer being referred to. Combined with the findings from Zheng et al. (2017), we can now sketch a more complete picture of what is likely to happen to the perceptual information in mental simulations. Specifically, perceptual features activate when they are consistently being implied in a narrative (Zheng et al., 2017), but does not remain activated when a reader’s attention is shifted away from those features. As such, the role of mental simulations in language comprehension seems to be for the purpose of activating targeted perceptual features.

One notable limitation of this study is that, although we examined the activation of color in an arguably more naturalistic context than single-sentence studies, an experiment using five sentences can still be considered impoverished compared with texts occurring in real life (Graesser, Millis, & Zwaan, 1997). As such, the generalizability of these findings to discourse processing as a whole is somewhat limited.

In conclusion, the current study has illustrated that color remains active in mental simulations so long as the target object is present in every sentence. As soon as the focus of the story shifts to another object, this perceptual information is deactivated in the mental simulation. As such, there is no continued activation of color across a broader discourse context. We started this article by referring to the color of Gandalf’s cloak. What makes this example different from the stimuli in our experiments is that the color change of the cloak is thematically relevant. It marks the transition from *Gandalf the Grey*, a somewhat cranky and eccentric figure, to *Gandalf the White*, the most powerful wizard of Middle Earth. It is clear that the color changes in our studies do not have such momentous implications. The study of whether and how such thematically relevant perceptual changes are represented by the comprehender is beyond the scope of this article but is an interesting topic for future research.

## Appendix 1

For all analyses reported in this Appendix, we used R (R Core Team, 2020) and the package lme4 (Bates, Maechler, Bolker, & Walker, 2015) to perform linear and logistic mixed-effects models. We obtained *p* values using a likelihood ratio test, as recommended by Winter (2013). The R code used for the analyses can be found in Appendix 2.

### Experiment 1

#### Accuracy

A logistic mixed-effects model was performed on the accuracy scores of Experiment 1. We entered the variables “sentence” and “picture” (with the interaction term) as fixed effects into the model using a manual step-wise step-up forward elimination procedure. We entered item and subject as random intercepts into the model. In the first step, only “sentence” was entered into the model to compare it with the null model with only the random effects, which caused a significant change in AIC and BIC scores, χ^2^(1) = 4.60, *p* = .032. As such, the variable “sentence” significantly improved the model fit and was kept in the model. In the second step, we added “picture” as a fixed factor to the model, which also significantly improved model fit, χ^2^(1) = 4.37, *p* = .037. Finally, we added the interaction effect (Sentence × Picture) as a fixed effect, which did not significantly improve model fit compared with when only the two main fixed effects were included, χ^2^(1) = 2.33, *p* = .127.

#### Response times

We performed a linear mixed-effects model on the RTs of Experiment 1. Similar to the logistic mixed-effects model, we used a step-wise step-up forward elimination procedure here, using a likelihood ratio to obtain *p* values. We entered item and subject as random intercepts into the model, with “sentence” and “picture” as the fixed effects (including the interaction term). In Step 1, we added only “sentence” to the model, which did not significantly improve the model, χ^2^(1) = 2.00, *p* = .157. As such, this variable was not included in the model. In Step 2, we added the variable “picture” to the model, which caused a significant reduction in AIC and BIC scores, χ^2^(1) = 12.75, *p* < .001, and therefore improved the fit of the model. In Step 3, we added the interaction term (Sentence × Picture) to the model, which also significantly improved the model fit, χ^2^(2) = 25.01, *p* < .001.

### Experiment 2

#### Accuracy

We performed a logistic mixed-effects model on the accuracy scores of Experiment 2 using the same method as for Experiment 1. In Step 1 we entered “sentence” as a fixed factor in the model and compared it to the null model (including only the random effects). “Sentence” did not significantly improve the fit of the model, χ^2^(1) < 0.001, *p* = .981, and so was not included as a factor in the subsequent steps. In Step 2, we added “picture” as a fixed factor to the model, which significantly improved the model fit, χ^2^(1) = 6.36, *p* = .012. In the final step, we added the interaction term to the model (Sentence × Picture), which did not significantly improve the model fit, χ^2^(2) = 1.36, *p* = .506.

#### Response times

We performed a linear mixed-effects model on the RTs of Experiment 2 using the same analysis method as for Experiment 1. Entering “sentence” as a fixed factor to the model did not significantly improve model fit, χ^2^(1) = 0.70, *p* = .402, and therefore was left out of the model in the subsequent steps. In Step ,2 we added “picture” as a fixed effect, which significantly improved the model fit, χ^2^(1) = 17.54, *p* < .001. In Step 3, we added the interaction effect (Sentence × Picture) to the model used in Step 2, which did not significantly improve the model fit, χ^2^(2) = 1.27, *p* < .529.

### Experiment 3

#### Accuracy

Identical to the analyses from Experiments 1 and 2, we performed a logistic mixed-effects analyses on the accuracy scores. In Step 1, we added “sentence” as a fixed effect, which did not significantly improve the model fit, χ^2^(1) = 0.02, *p* = .875, and which was not included in the subsequent steps of the model. In Step 2, we added “picture” as a fixed effect to the model, which significantly improved the model fit, χ^2^(1) = 4.26, *p* = .039. In the final step, we added the interaction between “picture” and “sentence” to the model, which did not significantly improve the model fit, χ^2^(2) = 4.00, *p* = .135.

#### Response times

We performed linear mixed-effects analysis on the RTs of Experiment 3 using the same method as for Experiments 1 and 2. In Step 1, we added “sentence” as a fixed effects factor to the model, which did not significantly improve the model fit, χ^2^(1) = 0.01, *p* = .911, and was therefore excluded from the model in the subsequent steps. In Step 2, we added the variable “picture” as a fixed effect, which also did not significantly improve the model fit, χ^2^(1) = 0.52, *p* = .472, and was therefore excluded from the model in Step 3. In Step 3, we entered the interaction between “sentence” and “picture” into the model, which significantly improved the model fit, χ^2^(3) = 11.12, *p* = .011.

## Appendix 2

The R code used to conduct the analyses mentioned in Appendix 1 are shown below. The packages used for the analyses are *stringr* (Wickham, 2019), *dplyr* (Wickham, Francois, Henry, & Müller, 2020), *tidyr* (Wickham & Henry, 2020), and *lme4* (Bates et al., 2015).

## References

[CR1] Barsalou LW (1999). Perceptual symbol systems. Behavioral and Brain Sciences.

[CR2] Barsalou LW (2008). Grounded cognition. Annual Review of Psychology.

[CR3] Barsalou LW, Santos A, Simmons WK, Wilson CD, de Vega M, Glenberg A, Graesser A (2008). Language and simulation in conceptual processing. Symbols and embodiment: Debates on meaning and cognition.

[CR4] Bates D, Maechler M, Bolker B, Walker S (2015). Fitting linear mixed-effects models using lme4. Journal of Statistical Software.

[CR5] Ditman T, Holcomb PJ, Kuperberg GR (2008). Time travel through language: Temporal shifts rapidly decrease information accessibility during reading. Psychonomic Bulletin & Review.

[CR6] Dove G (2016). Three symbol ungrounding problems: Abstract concepts and the future of embodied cognition. Psychonomic Bulletin & Review.

[CR7] Glenberg AM, Meyer M, Lindem K (1987). Mental models contribute to foregrounding during text comprehension. Journal of Memory and Language.

[CR8] Graesser AC, Millis KK, Zwaan RA (1997). Discourse comprehension. Annual Review of Psychology.

[CR9] Hoeben Mannaert LN, Dijkstra K, Zwaan RA (2017). Is color an integral part of a rich mental simulation?. Memory & Cognition.

[CR10] Hoeben Mannaert LN, Dijkstra K, Zwaan RA (2019). How are mental simulations updated across sentences?. Memory & Cognition.

[CR11] Kiefer M, Pulvermüller F (2012). Conceptual representations in mind and brain: theoretical developments, current evidence and future directions. Cortex.

[CR12] Levine WH, Klin CM (2001). Tracking of spatial information in narratives. Memory & Cognition.

[CR13] Morrow DG, Greenspan SL, Bower GH (1987). Accessibility and situation models in narrative comprehension. Journal of Memory and Language.

[CR14] Nagai JI, Yokosawa K (2003). What regulates the surface color effect in object recognition: Color diagnosticity or category. Technical Report on Attention and Cognition.

[CR15] Pecher D, van Dantzig S, Zwaan RA, Zeelenberg R (2009). Short article: Language comprehenders retain implied shape and orientation of objects. Quarterly Journal of Experimental Psychology.

[CR16] Pollatsek A, Well AD (1995). On the use of counterbalanced designs in cognitive research: A suggestion for a better and more powerful analysis. Journal of Experimental Psychology: Learning, Memory, and Cognition.

[CR17] Radvansky GA, Copeland DE (2006). Walking through doorways causes forgetting: Situation models and experienced space. Memory & Cognition.

[CR18] R Core Team (2020). R: A language and environment for statistical computing [Computer software].

[CR19] Rinck M, Bower GH (1995). Anaphora resolution and the focus of attention in situation models. Journal of Memory and Language.

[CR20] Speer NK, Zacks JM (2005). Temporal changes as event boundaries: Processing and memory consequences of narrative time shifts. Journal of Memory and Language.

[CR21] Stanfield RA, Zwaan RA (2001). The effect of implied orientation derived from verbal context on picture recognition. Psychological Science.

[CR22] Sundermeier BA, Van der Broek P, Zwaan RA (2005). Causal coherence and the availability of locations and objects during narrative comprehension. Memory & Cognition.

[CR23] Swallow KM, Zacks JM, Abrams RA (2009). Event boundaries in perception affect memory encoding and updating. Journal of Experimental Psychology: General.

[CR24] Tanaka JW, Presnell LM (1999). Color diagnosticity in object recognition. Perception & Psychophysics.

[CR25] Therriault, D. J., Yaxley R. H., & Zwaan, R. A. (2009). The role of color diagnosticity in object recognition and representation. *Cognitive Processing, 10*(4), 335-342. 10.1007/s10339-009-0260-4 10.1007/s10339-009-0260-4 19471986

[CR26] Tolkien, J. R. R. (2005). *The lord of the rings* (50th anniversary ed.). London, England: HarperCollins.

[CR27] Van Dijk TA, Kintsch W (1983). *Strategies of discourse comprehension*.

[CR28] Wickham, H. (2019). *Stringr: Simple, consistent wrappers for common string operations.* R package version 1.4.0. https://CRAN.R-project.org/package=stringr

[CR29] Wickham, H. & Henry, L. (2020). *Tidyr: Tidy messy data.* R package version 1.0.2. https://CRAN.R-project.org/package=tidyr

[CR30] Wickham, H., François, R., Henry, L., & Müller, K. (2020). *Dplyr: A grammar of data manipulation.* R package version 0.8.5. https://CRAN.R-project.org/package=dplyr

[CR31] Winter, B. (2013). *Linear models and linear mixed effects models in R with linguistic applications*. ArXiv Preprint. arXiv:1308.5499

[CR32] Yaxley RH, Zwaan RA (2006). Simulating visibility during language comprehension. Cognition.

[CR33] Zheng L, Huang P, Zhong X, Li T, Mo L (2017). Color adaptation induced from linguistic description of color. PLOS ONE.

[CR34] Zwaan RA (1996). Processing narrative time shifts. Journal of Experimental Psychology: Learning, Memory, and Cognition.

[CR35] Zwaan RA (2016). Situation models, mental simulations, and abstract concepts in discourse comprehension. Psychonomic Bulletin & Review.

[CR36] Zwaan RA, Langston MC, Graesser AC (1995). The construction of situation models in narrative comprehension: An event-indexing model. Psychological Science.

[CR37] Zwaan RA, Madden CJ, Yaxley RH, Aveyard ME (2004). Moving words: Dynamic representations in language comprehension. Cognitive Science.

[CR38] Zwaan RA, Magliano JP, Graesser AC (1995). Dimensions of situation model construction in narrative comprehension. Journal of Experimental Psychology: Learning, Memory, and Cognition.

[CR39] Zwaan RA, Pecher D (2012). Revisiting mental simulation in language comprehension: Six replication attempts. PLOS ONE.

[CR40] Zwaan RA, Radvansky GA, Hilliard AE, Curiel JM (1998). Constructing multidimensional situation models during reading. Scientific Studies of Reading.

[CR41] Zwaan RA, Stanfield RA, Yaxley RH (2002). Language comprehenders mentally represent the shapes of objects. Psychological Science.

